# "The Hospital" Medical Book Supplement.—No. XIII

**Published:** 1908-12-19

**Authors:** 


### "THE HOSPITAL" MEDICAL BOOK SUPPLEMENT.—No. XIII.

CONTENTS.

**Medicine—**
A System of Medicine ... 305
Minor Maladies and their Treatment... 305
**Surgery—**
The Rectum: Its Diseases and Developmental Defects ... 306
Third Scientific Report of the Imperial Cancer Research Fund 306
**Neurology—**
Quain's Elements of Anatomy... 307
Neurasthenia ... 307
**Dermatology—**
Diseases of the Skin ... 307
**Public Health and Hygiene—**
Principles and Methods of Physical Education and Hygiene... 308
The Principles of Hygiene as applied to Tropical and Sub-Tropical Climates ... 308
**Miscellaneous Items—**
Nelson's Shilling Library ... 308
The Matron: Her Duties and Responsibilities ... 308

### MEDICINE.

A System of Medicine. Edited by William Osler and Thomas McCrae. Vol. IV. Oxford Medical Publications. (London, 1908. Pp. 865. 24s. net per set of 7 vols., or 30s. per single copy.)

The fourth volume of Osler and McCrae's System of Medicine is devoted to diseases of the circulatory system, blood, spleen, thymus, and lymphatic glands. The various sections are treated with great thoroughness and reach a high level of excellence. The opening section, from the pen of Dr. Charles Hoover, is an interesting and, on the whole, explicit essay upon the general considerations which govern the normal and abnormal functions of the heart. It contains a review of the recent theories of cardiac activity and supplies an excellent introduction to the study of cardio-vascular disease. Dr. Hoover is also responsible for the section concerned with functional heart disease, another good piece of work. He emphasises (what is so slowly yet so certainly brought home to the observant clinician) the fact that "organic" and "functional" diseases merge into one another, as in the case of angina pectoris, and that our present conception of functional disease of the heart is not at all inconsistent with heart-death as a consequence. We can strongly recommend this chapter to the student of heart-disease, for there is no doubt that in our schools all the multitudinous aberrations of the heart which are not associated with gross manifestations of valvular insufficiency meet with a wholly undeserved neglect. This is due in part at least to the facts that our knowledge of them is still in its infancy, and that the teacher of elementary students must be dogmatic, but it is to be regretted. Dr. A. McPhedran supplies the chapter upon the pericardial diseases. It is well done, but we think that enough stress is not laid upon the difficulties of the diagnosis of purulent pericarditis in young children. In this country purulent pericarditis due to the pneumococcus, and affecting young children, is not by any means a great rarity, and its diagnosis is a most difficult problem. For the effusion is often small and may be more or less loculated behind the heart, thus obscuring the ordinary signs of pericardial effusion. Dr. Robert Babcock's chapter upon the diseases of the myocardium is complete and good. The detailed description of various means of treating myocardial insufficiency is worthy of imitation in text-books of medicine, for the directions for treatment are often vaguely given and leave the reader in doubt as to whether or not the author has copied them out of some other book. Professor Osler himself deals with acute endocarditis, diseases of the arteries, and aneurysm. It is hardly necessary to say that these sections are careful and thorough. But a quite unusual merit attaches to that upon diseases of the arteries, for here we have an intelligible and comprehensive view of these diseases in their many aspects, rather than the bewildering enumeration of names, descriptive of anatomical variants, which has made this subject a byword for difficulty to the student. Congenital anomalies of the heart are exhaustively dealt with by Dr. Maude E. Abbott. The only adverse criticisms of this treatise which we have to offer are, firstly, that some of the photographs of anomalies seem so obscure as not to repay reproduction, and secondly, that the explanation offered for the physical signs of patency of the ductus arteriosus does not seem to us very convincing. Dr. Alexander Gibson treats of hypertrophy of the heart, and of insufficiency and dilatation, chiefly from a theoretical point of view. The inclusion of such essays as these is in itself evidence of the exhaustive character of the work. The chapter on valvular disease in general is the joint labour of Professor Osler and Dr. Gibson. Here again we have to observe with satisfaction that the treatment is given in sufficient detail to be of real practical service. The greater part of the section dealing with diseases of the blood is from the pen of Dr. Richard Cabot, whose reputation in this field is surety for the excellence of his contributions. In the article upon leukæmia Dr. Cabot urges the essential unity of leukæmias, whether clinically myeloid or clinically lymphatic. That is to say, the leucocytic hyperplasia is the essence of both diseases, and this may begin in the glands, spleen, or marrow, without indicating any fundamental difference in the underlying process. The primary anæmias are so mysterious that any authoritative generalisation is a welcome sign of the gradual correlation of data hitherto isolated and confusing.

Minor Maladies and their Treatment. By Leonard Williams, M.D., M.R.C.P. (London: Baillière, Tindall, and Cox. Second edition. Price 5s. net.)

If ever there was a medical book essentially for the intelligent general practitioner, it is this manual of Dr. Williams, a second edition of which is now published. The first edition has gained such wide popularity that it is hardly necessary now to notice more than the chief alterations and additions which have been made. The most important of these is perhaps the considerable length at which the subject of "pretuberculosis" is now discussed at the end of the chapter on coughs and colds; though we do not feel quite certain that the signs allotted to this elusive condition by the French physicians mean always quite what they are believed to. In the sections on "Some Drugs and their Uses" much recent work is included, as is indeed the case throughout the volume. There are two features to which particular attention may be drawn, because deficiencies in respect of them are so woefully common in medical literature. One is the scholarly style, and consequent lucidity; the other is the very unusually full index. The reputation which "Minor Maladies" has already rapidly acquired will certainly be augmented by this, its second edition.

### SURGERY.

The Rectum : Its Diseases and Developmental Defects. By Sir Charles B. Ball. 8vo. Pp. 328, fully illustrated. (London : Hodder and Stoughton, and Henry Frowde. 1908. 30s. net.)

In 1902 Sir Charles Ball delivered the Lane lectures in San Francisco on "Diseases of the Rectum," and the following year the Erasmus Wilson lectures on "Adenoma and Adeno-carcinoma of the Rectum." He has now replaced his former work on the rectum and anus by the present handsome volume, which embodies the material collected for the lectures and makes a complete, if unequally balanced, monograph on the subject. The student and general reader not in search of statistics will welcome a book readable from page to page and from cover to cover, copiously and remarkably well illustrated, notable, too, for its typography. The surgeon will naturally turn to the chapter on the operative treatment of cancer of the rectum, and to a certain extent he will be disappointed. A laudable desire to be up to date has led Sir Charles to detail, not his own great experience, but the latest developments of combined abdominal and perineal or sacral operations, and even to devise an anatomically perfect method for aseptic excision by the posterior route. The latter, we are told, works out well in the deadhouse, but Sir Charles has had but one opportunity of performing it *intra vitam*, and we confess to some doubt as to the feasibility of such anatomical exactitude as his method requires for success. It is to be noted that the author does not approve of colotomy as a preliminary to excision of the rectum, if there is any idea of making a permanent posterior anus. The method of treating piles is a combination of the ligature and crushing; the description is lucid and minute, and the diagrams excellent; we believe there is no method so satisfactory as this when carried out with proper attention to detail. The estimate of the value of Whitehead's operation is sound, for it is based on complete appreciation of the anatomical considerations involved. This leads us to remark that throughout the book the most striking feature is the elaborate care with which anatomical details are described and made the basis of treatment. The chapters on developmental defects give a succinct account of the chief varieties, but, perhaps wisely, treatment is dismissed in a page or two. The author has a good deal to say about both tuberculosis and syphilis, under the heading "Infective Diseases," that will be read with interest. In the treatment of fistula, Stephen Smith's plan of sharp-spooning, disinfecting, and suturing the track after it has been laid open, is recommended, and is stated to give very satisfactory results. In speaking of stricture Sir Charles brings forward a case to support the view of Harrison Cripps that a condition of intermittent spastic contraction of the muscular coats may result in permanent shortening of the muscular fibres and, with increase of the connective tissue elements, in the production of stricture. To describe the peculiar character, duration, and initiation of the pain of a fissure, the familiar analogy of a hang-nail or "torment," as it is called here, is suggested. So soon as the little piece of loose skin is cut away the pain ceases; in like manner the painful ulcer, due, as here claimed, to the tearing down of one of the anal valves, will cease to torture and heal up without difficulty if only the tag of skin be removed. In other words, treatment of the so-called "sentinal pile" is all that is required. In the treatment of inveterate prolapse, Lenormant's operation is advised; it is precisely similar to that practised for retroperitoneal or "gliding" herniæ of the cæcum, and involves denudation of peritoneum from an area of iliac fascia parallel to but outside the left ureter; the pelvic colon being pulled up is sutured to the peritoneal edges of the incision and strong sutures are passed through the iliacus and one of the longitudinal bands. The chapter on innocent tumours is well worth reading. To those interested in rectal surgery, and not in search of a cumbersome treatise, we can recommend this book as thoroughly practical and most instructive.

Third Scientific Report of the Imperial Cancer Research Fund. By Dr. E. F. Bashford. (London : Taylor and Francis. 1908. Pp. 483. Price 15s.)

A detailed consideration of this report, to which we made a brief allusion upon its appearance, confirms the favourable impression derived at that time. It is a monument of diligence and of a persevering thoroughness which augurs well for the future progress of knowledge with respect to cancer. One of the great objects of the report being the provision of means whereby the results obtained by the researchers of the fund may be confirmed by other workers, the minutiæ of the technique in vogue for each series of experiments are given in detail. It is this fact which accounts for the bulk of the volume, and for the highly technical character of the greater part of its contents. It is indeed a pure text-book on the subject, and does not pretend to cater for the curiosity of the public, however deeply the interests of this body is involved in the progress of cancer-research. So much is this the case that one may say there are only two points emphasised by the contributors which can at present be appreciated by everybody. These are, firstly, that cancer is not, as used at one time to be thought, the melancholy prerogative of man, but is shared by all vertebrates, except perhaps the reptiles; secondly, that the artificial propagation of new growths in mice confutes the belief that heredity plays a part in the incidence of cancer. Up to the present the in-breeding of carcinomatous mice has not enhanced the liability of their offspring to develop carcinoma. As regards the technical matter in the volume we cannot here do more than make the briefest references to a few of the more prominent items of advance. Dr. Murray has shown the falsity of the commonly-heard assertion that the malignancy of tumours is enhanced by surgical interference with them. Dr. Haaland, in a most exhaustive communication, deals at length with the development of sarcomata during the experimental propagation of carcinomata. It appears to be demonstrated that in the course of propagation the stroma elements of a mouse carcinoma may gradually acquire the proliferative characters of malignancy, this enhancement of their growing powers leading ultimately to the extinction of the carcinomatous elements and the final conversion of what was a carcinoma into a pure sarcoma. The bearing of this important observation is not at present clear, but it is obviously of great moment in the biological study of malignant growths. Drs. Bashford, Murray, and Cramer supply an interesting section upon the natural and induced resistance of mice to the growth of cancer. It appears that mice in which transplantation has been effected, but in which the transplanted tumour has subsequently disappeared spontaneously, are rendered extremely refractory to subsequent inoculations of the same growth. It is presumed that the absorption of the tumour elements has endowed the host with a specific resistance of which it was not previously possessed. This experiment cannot fail to raise hopes of some therapeutic application, but we are, very wisely, warned that such expectations are yet untimely. The whole volume is profusely illustrated with excellent micro-photographs and charts, which cannot fail to be of the utmost service to all subsequent researchers.

### NEUROLOGY.

Quain's Elements of Anatomy, in four volumes. Vol. III. Part I., Neurology. By E. A. Schäfer and J. Symington. Eleventh edition. (Longmans, Green and Co. 1908. Royal 8vo. Pp. 421. 15s. net.)

The appearance of a new edition of this, the most authoritative English text-book, must be regarded as a landmark in the anatomical world. The eminence of the joint authors is a guarantee that the high standard of the previous editions would be amply maintained, and this hope is fully justified by a study of the present volume. The part now published discusses the general structure and mode of development of the cerebro-spinal and sympathetic nervous systems, followed by a detailed account of the anatomy of the cerebro-spinal axis. It is shortly to be succeeded by a second part embracing the descriptive anatomy of the peripheral nerves and of the sense organs, the two parts together constituting a complete text-book of neurological anatomy. It is impossible within the limits of a brief review to give an adequate account of the wealth of facts lucidly set forth. The text is illuminated by over 360 figures, both original and borrowed from other sources. It is interesting to note that the editors still regard the neurone theory, "even should the doctrine of discontinuity be disproved," as established, in spite of the trenchant criticisms which have been levelled against it by competent observers. Surely the neurone theory, shorn of the dogma that each neurone is morphologically independent of all other neurones, is like "Hamlet" without the Prince of Denmark? On another disputed point, the mode of regeneration of nerve-fibres, the editors declare themselves in favour of the central theory, and they discard the opposing view as to the possibility of regenerative processes occurring in the peripheral segment of a divided nerve. But, after all, it is the business of a text-book to be dogmatic, and from the student's point of view it is better so. For the account of the naked-eye and microscopic anatomy of the spinal cord and brain we have nothing but praise. We would call special attention to Professor Symington's excellent series of new drawings of the external conformation of the cerebellum and cerebrum. He presents views of the cerebrum from the lateral aspect, from above, from behind, and from the mesial aspect. There is a most valuable section of cranio-cerebral topography, with an orthogonal projection of the medulla, pons, mid-brain and visual tracts in one diagram, and with projections of the cerebral ventricles in others. Elliott Smith's diagrams of naked-eye appearances of sections of the fresh cerebral cortex are reproduced. The book also contains the best account we have yet read of the anatomy and relations of the cranial dura mater; this part being illustrated by several most instructive figures. The work is the best one on anatomy at present existent in the English language, and we look forward with interest to the appearance of the second part of the volume.

Neurasthenia. By Gilbert Ballet, Professor agrégé à la Faculté de Médecine de Paris. Translated from the third French edition by P. Campbell Smith. (London: Henry Kimpton, 1908. Octavo, pp. 408, with seven figures. Price 6s. net.)

This is one of the best known French works on neurasthenia. The first two editions of the book were written conjointly by Professors Ballet and Proust. The third edition is by Ballet alone, and it is this latter which is now presented to English readers in an admirable translation by Dr. Campbell Smith. The book is a practical treatise by an acute clinician and well repays study. A clear and vivid account is presented of the different clinical varieties of neurasthenia. It is interesting to note that Ballet does not accept the modern clinical differentiation between psychasthenia and neurasthenia, a distinction which has recently been so emphatically urged by Raymond and others. He prefers to speak of congenital or "precocious" neurasthenia, and somewhat unfortunately, we think, labels it "neurasthenia minor." With this exception the subject-matter of the work is excellent. Ballet regards neurasthenia as a somatic disease, in this respect contrasting it with hysteria. He emphasises the importance of over-pressure as an exciting cause, whilst not forgetting the important rôles played by neuropathic heredity, by toxic conditions, and by traumata, physical or mental. The symptoms and clinical forms of neurasthenia are exhaustively discussed, the author recognising four main varieties of the disease—cerebro-spinal, traumatic, genital, and the neurasthenia of women. As to the prophylaxis of neurasthenia, he gives an interesting discussion of the physical and moral educational lines on which children should be brought up who are predisposed by heredity to nervous exhaustion. In the treatment of the actual disease he gives elaborate descriptions of pschyo-therapeutics, of diet, of hydro-therapeutics, of climatic treatment, and of suitable exercises and gymnastics. Special chapters are devoted to the treatment of the neurasthenia of women (a condition specially suitable for the Weir-Mitchell system of isolation, diet, massage, and electro-therapeutics), and to the management of cases of genital neurasthenia.

### DERMATOLOGY.

Diseases of the Skin. By Sir Malcolm Morris, K.C.V.O. New edition, revised by the author and by S. E. Dore, M.D., M.R.C.P. (London: Cassell and Co., Limited. Price 10s. 6d.)

This book has long held an established position among that select list of special monographs of which no medical man who is keen on his profession omits the perusal. It belongs to the same class as, for instance, the late Sir Wm. Broadbent's book on heart disease, in that it contains everything that is essential for a non-specialist to know, and is overloaded with little or nothing that is irrelevant. Since the first edition was published in 1893 there have been five new editions and reprints, not including this, which is termed the fourth edition; and the very great progress which has been made, especially during the last decade, in our knowledge of skin diseases is well shown by the wide difference between the first edition and the present one. To criticise in detail a work so well known as this is needless, and would indeed be difficult. If there is one way in which we could wish the system on which the volume is compiled changed, it is by the omission of a certain number of the opinions, researches, and dogmas of various authors whose work has failed to stand the test of time. That is to say, we think Sir Malcolm here and there is a little too catholic and too prone to quote the views of other dermatologists, views which are in some cases to all intents disproved. We observe with interest that the parasite of syphilis is still termed *spirochœta pallida*, notwithstanding the efforts of certain authorities to have adopted the alternative *treponema pallidum*. Whilst on this subject it may be mentioned that there is no mention of Metchnikoff and Maisonneuve's method of prophylaxis. The photographs and coloured plates, which now amount to over eighty, are for the most part excellent; the steady improvement of the text since the first edition is equalled, if not excelled, by that of the illustrations, which enhance the value of the letterpress very greatly.

### PUBLIC HEALTH AND HYGIENE.

Principles and Methods of Physical Education and Hygiene. By W. P. Welpton, B.Sc. (London : W. B. Clive, 1908. Price 4s. 6d.)

A very large part of this text-book is occupied by an exposition of the various elements of anatomy, physiology, and pathology which bear upon the matter under discussion. Almost without exception this medical information is accurate, as far as it goes, and germane. It is, moreover, free from the reproach which attaches to so many efforts to disseminate medical knowledge among the laity, that of tending towards the dangerous little knowledge of disease and cure that ends in self-drugging and other evils. The close supervision which has been exercised by a physician (Dr. O. T. Williams) probably explains this satisfactory result. At the same time we do distinctly share the fear expressed in the preface that some of the physiological descriptions may be difficult of comprehension for ordinary readers, whom the author advises to do a little judicious "skipping." For those who can fully understand the whole text, there are obviously great advantages in laying bare the chain of reasoning by which the principles of physical hygiene are anchored. So, too, the historical account of the progress and tendencies of education from the earliest times is of very great interest and value : this is contributed by Professor Welton, and is both exhaustive and compact. In the arrangement of the subsequent chapters a logical sequence is preserved by dealing first with the physical basis of life and such elementary physiology as is necessary to its understanding; then with bodily hygiene and the methods for promoting it not only by education, but also during the educative process; and lastly, with the pathological aspects of school life. The subject is one of the greatest possible moment, and one whose treatment at Mr. Welpton's hands is eminently satisfactory.

The Principles of Hygiene as applied to Tropical and Sub-Tropical Climates. By W. J. R. Simpson, M.D., F.R.C.P., D.P.H., formerly Health Officer of Calcutta, Lecturer on Tropical Hygiene at the London School of Tropical Medicine; Professor of Hygiene, King's College. (London : Bale, Sons, and Danielson.)

Dealing with a subject that is interesting at home and even more so abroad, Professor Simpson contributes an exceedingly interesting manual to the literature of public health. Hygiene must of necessity be somewhat different in cold and warm climates, owing to the part insects play in the dissemination of disease, to the habits of the different native races, to the temperature, to the food, and so on, and special stress is laid on these points. The author has had a varied and prolonged experience of India and other tropical colonies, and the pages of the book are really his own personal observations and matured experience. The work, with the index, extends to 396 pages, and gives a very full digest of the whole subject. The only faults to find are that some of the parts might with advantage have been fuller still, but space, no doubt, was an object when this had to be considered. A special feature of the work is the excellence of the diagrams and plans which illustrate the book. These greatly help the due appreciation of the different methods detailed. It will be well worth anyone's time to get a copy of the manual; certainly all going abroad should read it carefully.

### MISCELLANEOUS ITEMS.

The Matron : Her Duties and Responsibilities. (The Scientific Press, London, W.C. Price 2s. 6d. net.)

This volume should be most helpful to nurses who aspire to take up the duties of matron either in a large or a small hospital, as well as to matrons who desire promotion; for hitherto there has been no book to which either could look for guidance. Although it is not possible in the space of 123 pages to state all that a matron should be familiar with before taking up her duties, it is astonishing how much valuable information is contained in this volume, which fills a distinct want. Its perusal will make it perfectly clear why it is that the matron should be a lady in the best sense of the word. No matter how skilful a woman may be or how well she is versed in the theory and practice of nursing, if she is not possessed of all the instincts of a lady she can never hope to be a successful matron. A lady at the head of an institution always knows instinctively her limitations. Such a matron confines herself strictly to her department and works harmoniously with the medical staff, and so is respected by her subordinates. These points are properly made, and the importance of great self-control and discipline is wisely insisted upon. "Every hour gives opportunity for self-restraint, patience, and courage in dealing with subordinates, for loyalty and sincerity in obeying superiors, for reticence and the exercise of a judgment unbiassed by personal slights and favours." It is for this reason that it is so difficult to find suitable candidates of the best type to fill responsible posts in the nursing world. In the United States infinite pains are taken to give practical instruction in the duties of a matron, and some of the larger schools in this country have arranged in recent years to give similar instruction. It is essential to the successful working and advance of the higher nursing in this country that those responsible for the training of nurses should take upon themselves the duty of ascertaining as far as possible which of their certificated nurses have the qualifications necessary for higher offices in the hospital world. If the requisite qualities are non-existent in the nurse they will never probably be acquired. We should therefore like to see provision made in every nurse-training school for the higher education of the more intelligent probationers in the duties of a matron and the institution of an additional certificate, or a special certificate, which would testify when a nurse is found to possess the essential qualities required for the higher appointments. This book contains the reasons and illustrates the necessity for this new departure on the part of nurse-training schools. It should be carefully read and studied by all who have to do with the training of nurses as well as by all who aspire to the office of matron or who have directly or indirectly to do with her department. A matron to be succesful, as the author insists, should be quick to see the good points in others and slow to condemn, for so she meets with the maximum success in the government of her institution.

Nelson's Shilling Library.—We have received from Messrs. Thomas Nelson and Sons, the well-known publishers of cheap and tasteful editions, the first four volumes of their new "Shilling Library"—a series of reprints of copyright works on travel, biography, history and other literary subjects. The four volumes sent to us are respectively Edward Whymper's *Scrambles amongst the Alps*; *Collections and Recollections*, by the Right Hon. G. W. E. Russell; Col. Trotter's *Life of John Nicholson*; and Sir A. Conan Doyle's *The Great Boer War*. The series is attractively got up, and uniform in size with Nelson's "Sixpenny Novels."

